# Morin Improves the Bone Histomorphology and Biochemical Markers in an Animal Model of Ovariectomy‐Induced Osteoporosis by Suppressing Autophagy and Apoptosis

**DOI:** 10.1002/fsn3.4554

**Published:** 2024-10-28

**Authors:** Nan Jiang, Bo Qi, Gang Li, Ling Yao, Xinyu Fan

**Affiliations:** ^1^ Department of Orthopedics 920th Hospital of Joint Logistics Support Force Kunming China

**Keywords:** apoptosis, autophagy, morin, osteoporosis, ovariectomy

## Abstract

Osteoporosis (OP) is the most prevalent metabolic bone disease and an important postmenopausal consequence. This study aimed to investigate the effects of morin, a flavonoid with beneficial properties, on ovariectomy‐induced OP. Animals were ovariectomized (OVX) and treated with different doses of morin (15, 30, and 45 mg/kg/day) or estradiol (10 μg/kg/day) for 10 weeks by gavage. Then bone histo‐stereology, bone‐related biochemical indicators, and gene and protein levels of autophagy and apoptosis‐related markers were analyzed. In comparison to controls, OVX significantly decreased the number of osteoblasts (5.78 × 10^6^ vs. 1.66 × 10^6^) and osteocytes (32.55 × 10^6^ vs. 11.92 × 10^6^), whereas increasing the number of osteoclasts (83.38 × 10^3^ vs. 392.1 × 10^3^). Moreover, OVX caused a remarkable decrease in bone structures and Ca, P, and estradiol levels while increasing ALP and OC (*p* < 0.001). The administration of 45 mg/kg/day morin restored the effects of OP on bone histomorphology and biochemical markers (*p* < 0.05). Further studies revealed that morin caused a 7.1% and 36.6% decrease in the bone level of LC3 and BECN1 proteins, respectively, compared to the OVX group. Also, morin caused a significant decrease of 47.4% in the CASP3 level and a significant increase of 23.6% in the BCL‐2 level compared to OVX animals (*p* < 0.001). The present findings showed that morin is potentially able to improve the bone‐related histomorphological and biochemical changes caused by osteoporosis, which is probably attributed to the suppression of apoptosis‐ and autophagy‐caused cell death.

## Graphical abstract

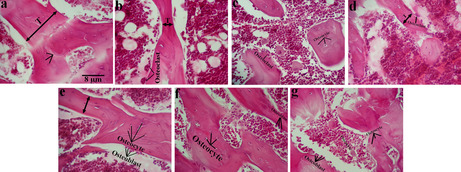

## 1 Background

Osteoporosis (OP) is a chronic disorder known as the most common skeletal pathological state that affects millions worldwide. It is characterized by a substantial decrease in bone density, leading to a weakening of the bone structure and disruption in the normal architecture of the skeletal system, which affects people of all ages, genders, and ethnic backgrounds (Langdahl 2021; Tu et al. 2018). OP leads to increased bone fragility, long‐term disability, reduced quality of life, and higher mortality rates, placing a significant economic burden on individuals and the public health system all over the world (Tu et al. 2018). The disease is often asymptomatic, only becoming apparent when a fracture occurs, making it challenging to diagnose and treat (Tu et al. 2018; Jeremiah et al. 2015). The underlying causes of OP are complex, involving a combination of genetic, hormonal, and nutritional factors, along with individual lifestyle. Nevertheless, estrogen (E2) deficiency after menopause is a key factor in bone loss and OP occurrence (Tella and Gallagher 2014; Klein‐Nulend et al. 2015).

Bone remodeling represents the biological process that ensures the ongoing renewal and repair of the skeletal framework. This complex procedure is characterized by the coordinated actions of osteoclasts, which are responsible for the breakdown of old bone material. Subsequently, osteoblasts are activated to produce collagen, thereby aiding in the generation of new bone tissue (Eriksen 1986). However, a disparity exists within this process where the rate of bone resorption exceeds the rate of bone formation, potentially resulting in bone depletion and the subsequent onset of OP (Shao et al. 2015). The complexity of the bone remodeling process has prompted ongoing research to elucidate the underlying molecular mechanism of the disease. Several studies have assumed the induction of oxidative stress and inflammation as key contributors to the occurrence and progression of OP (Mohamad, Ima‐Nirwana, and Chin 2020; Riegger et al. 2023). Moreover, recent research death, underscores the pivotal importance of autophagy and apoptosis in preserving bone health, as they serve as major regulators of cell survival and encompassing both osteoclasts and osteoblasts. Disruptions in these cellular processes have been linked to the development of OP (Florencio‐Silva et al. 2017; Li et al. 2020). It has been shown that when autophagy genes are knocked out in osteoblasts, the process of bone mineralization is disrupted (Pierrefite‐Carle et al. 2015; Greenhill 2016). This disruption has been observed in both human and animal studies, indicating that irregularities in autophagy can initiate bone loss, which in turn leads to OP (Pierrefite‐Carle et al. 2015; Onal et al. 2013). Concordantly, the importance of bone cell apoptosis in maintaining the health and functionality of bone tissue has been highlighted previously (Tella and Gallagher 2014). An increase in the apoptotic process among osteocytes and osteoblasts can lead to decreased bone formation, resulting in bone loss and the onset of osteopenia (Plotkin 2014).

Addressing OP involves a multifaceted approach, integrating both pharmacological and nonpharmacological methods (Gregson et al. 2022). The administration of E2 as part of hormone replacement therapy (HRT) remains the most potent method for confronting OP and other menopause‐related symptoms (Delmas 1997). However, concerns about the long‐term safety of current medications and the need for innovative therapeutic approaches underscore the importance of ongoing research and development (Tu et al. 2018; Potter et al. 2018). Efforts are ongoing to elucidate the molecular mechanisms involved in the emergence and progression of OP, as well as to evaluate novel therapeutic approaches. Recently, a plethora of studies have suggested herbs as novel complementary therapies, as these compounds provide favored properties such as antioxidant and anti‐inflammatory activities and the regulation of hormonal homeostasis and cell survival (Li et al. 2023; Mohapatra et al. 2024; Wang et al. 2024).

The involvement of intracellular signaling pathways and the recent interest of researchers in using nutrients in the treatment of chronic diseases have created a new topic known as nutrigenomics. For example, in the case of bone diseases, Naselli et al. (2023) have recently discussed that phytochemicals with antioxidant and anti‐inflammatory properties could modulate DNA interactions, which in turn may regulate hormonal homeostasis and cell survival (La et al. 2024). Morin hydrate, often simply referred to as morin, is classified as a flavonol, a specific type of flavonoid. This classification is unique due to its distinct flavone and flavonol rings, which set it apart from other plant compounds (Caselli et al. 2016). The comprehensive research on morin highlights its potential health benefits and therapeutic applications. Research has underscored the critical role of morin in numerous cellular functions, including inflammation, apoptosis, and autophagy. These effects are achieved through its interactions with multiple signaling pathways (Cakmak et al. 2023; Varışlı et al. 2022; Yesildag et al. 2022). In fact, previous studies have determined that morin is able to prevent OP and suppress the formation and function of osteoclasts by modulating MAPK, NF‐κB, and calcium signaling pathways (Shi et al. 2021; Wang et al. 2018). However, the effect of morin on regulated cell death programs in animal models of ovariectomized (OVX)‐induced OP is not elucidated.

OP has remained a major concern of the health system, although much attention has been paid to herbs as an innovative complementary treatment approach. This study aimed to explore the impact of morin on OVX rats, which are widely used as a model for postmenopausal OP research. By contrasting morin's effects with those of synthetic E2, the research aimed to elucidate morin's potential role in protecting bone health by examining bone histo‐stereology, bone biochemical markers, autophagy, and apoptosis.

## 2 Materials and Methods

### 2.1 Chemicals

Morin and E2 were obtained from Sigma Chemical Company. The levels of osteocalcin were measured by the commercial enzyme‐linked immunosorbent assay (ELISA) kits. (OC, Shanghai Crystal Day Biotech Company), B‐cell lymphoma 2 (BCL‐2), caspase 3 (CASP‐3), microtubule‐associated protein 1A/1B‐light chain 3 (LC‐3), and beclin‐1 (BECN1, mybiosource, Southern California, San Diego, USA). MyBioSource Company, USA, provided kits for evaluating serum levels of calcium (Ca), phosphorus (P), and alkaline phosphatase (ALP). The EZ RNA reagent and Revert Aid First Strand cDNA Synthesis Kit were supplied by Biobasic Inc. and Fermentase, respectively.

### 2.2 Experimental Design

In this research, 56 adult female Sprague–Dawley rats were included, which were randomly divided into seven distinct groups as follows: Group 1: Sham‐operated (Sham), Group 2: Ovariectomized animals with no intervention (OVX), Group 3: OVX animals treated with E2 vehicle (3% ethanol) (OVX‐ETH), Group 4: OVX animals treated with 15 mg/kg/day morin (OVX‐M15), Group 5: OVX animals treated with 30 mg/kg/day morin (OVX‐M30), Group 6: OVX animals treated with 45 mg/kg/day morin (OVX‐M45), Group 7: OVX animals treated with 10 μg/kg/day E2 (OVXE2).

All interventions were administered via gavage using bulb‐tipped gastric gavage needles (24 GA, 25 mm) over 10 weeks. The commencement of all treatments took place 1 week post‐ovariectomy. It should be noted that in this study, distilled water was used as a morin vehicle.

### 2.3 Ovariectomy Procedure and Sample Collection

The dorsal approach was performed for the bilateral ovariectomy procedure under the xylazine (Alfasan, Holland) and ketamine10% (BREMER PHARMA GMBH, Germany) anesthesia. Initially, after shaving and disinfecting the dorsal area of the animals using ethanol (70%), a precise 2 cm incision was made under sterile conditions, and the ovaries were successfully removed. The incision was carefully sutured, and the incision site was locally applied with lidocaine and tetracycline ointments to aid in the healing process and prevent infection (Vakili et al. 2020). After a treatment period of 10 weeks, the rats were euthanized under xylazine 2% and ketamine anesthesia, and blood samples were obtained through cardiac puncture. Under appropriate conditions for biochemical analysis, the extracted serum was preserved. Additionally, the tibia from each animal, free from cartilage, adherent tissues, and bone marrow, was stored at −80°C for RNA extraction and real‐time polymerase chain reaction (qRT‐PCR) analysis. For further histo‐stereological analysis, another tibia from each rat was preserved in 10% formaldehyde.

### 2.4 Histo‐Stereological Analysis

The samples preserved in 10% formaldehyde were used for histomorphological and stereological analysis of bone tissue. In this regard, the removed tissue was fixed in formalin 10%, processed, and blocked in cylindrical paraffin blocks. Serial sections with 5 μm thickness (10 sections per animal) were obtained by a microtome and stained with Hematoxylin and Eosin (H&E). A specialist histopathologist who was blinded to grouping conducted the histological analysis. Moreover, the stereological analyses were performed as described below.

#### 2.4.1 Assessment of Bone Cell Density and Quantity

Both the numerical density and the total count of bone cells were ascertained by the dissector method according to a previous study (Noorafshan et al. 2012) and using the following formula:

$$\mathrm{Nv}=\frac{\sum_{i=1}^{n}Q}{\left(\sum P*h*a/\text{frame}\right)}$$

where ⅀*Q* represented the total number of whole cells counted across all dissectors, *a*/frame was the area of the counting frame, *h* was the height of the optical dissector, ⅀*Q* was the total number of counted frames, *t* was the mean of the final section thickness, and BA was the microtome block advance to cut the block. The total count of bone cells was ascertained according to the following formula:

$$N\;\left(\text{bone cells}\right)=\mathrm{Nv}\;\left(\text{cells}/\text{bone}\right)\times V\;\left(\text{final}\right)$$

#### 2.4.2 Estimation of Bone Volume and Density of the Bone's Trabeculae Volume

The entire tibia was employed in the stereological analysis. The immersion method was employed to measure the primary volume, *V* (primary), of the tibia (Karbalay‐Doust, Noorafshan, and Pourshahid 2012). Following a prior research approach, the sections were meticulously organized using the orientator method (Mattfeldt et al. 1990). The degree of shrinkage (Dsh) was computed using the formula (Gundersen et al. 1988):

$$\text{Degree of shrinkage}\;\left(\mathrm{Dsh}\right)=1–\left(\text{area after}/\text{area before}\right)\;1.5$$

The “area after” referred to the surface area of each circular segment of the bone after it underwent processing and staining, whereas the “area before” denoted the surface area of each circular segment of the bones prior to these procedures. The total volume of the bone was determined using the following formula:

$$V\;\left(\text{final}\right)=\left(1-\mathrm{Dsh}\right)\times V\;\left(\text{primary}\right)$$

A video‐microscopy setup, comprising a Nikon E‐200 microscope (Japan), a Samsung SCB‐2000P digital camera (Korea), and a personal computer, was employed for this study. The volume density of trabeculae, denoted as Vv (trabeculae), which signifies the proportion of the unit volume of bone occupied by trabeculae, was determined utilizing the point counting technique based on the Delesse formula (Nyengaard 1999) applied to sections with a thickness of 4 μm:

$$\mathrm{Vv}\;\left(\text{trabeculae}/\text{bone}\right)=P\;\left(\text{Trabeculae}\right)/P\;\left(\text{reference}\right)$$

Here, P (Trabeculae) denotes the number of test points falling on the trabecular bone, and P (reference) (equal to P (total)) indicates the number of test points falling on the reference tissues (trabecular and bone marrow) of the tibia bone. The absolute trabecular volume was estimated using the following formula:

$$V\;\left(\text{bone}\right)=V\;\left(\text{final}\right)\times \mathrm{Vv}\;\left(\text{bone}\right)$$

### 2.5 Serum Markers Analysis

The ELISA kit was utilized for assessing the concentrations of serum OC, a key indicator of bone formation. The MyBioSource kits were employed to quantify the serum concentrations of Ca, P, and ALP, which were then processed through the Mindray BS200 autoanalyzer. Additionally, the serum 17β estradiol (E2) concentration was determined using commercially available ELISA kits, following the producer's instructions.

### 2.6 qRT‐PCR Assay

RNA was extracted from the total sample using the EZ RNA reagent as per the manufacturer's instructions. The 260/280 and 260/230 ratios of RNA were evaluated using the Biotek Nanodrop system. The integrity of the RNA was further assessed through 1.2% agarose gel electrophoresis. Complementary DNA (cDNA) was synthesized using the Revert Aid First Strand cDNA Synthesis Kit (Fermentase) with the purified RNA. Using SYBR Green on the Applied Biosystems 7500 System, the qRT‐PCR was performed. Initially, the samples underwent a denaturation phase for 5 min at 95°C. Following this, the samples were subjected to 35 cycles of amplification, with specific parameters for annealing (45°C for 45 s), elongation (72°C for 1 min), and denaturation (95°C for 45 s). To ensure the completion of reaction products, the duration of the final elongation reaction was extended to 10 min at 72°C. The expression levels of β‐actin, caspase 3 (CASP‐3), LC3, beclin1 (BECN1), and Bcl2 genes were assessed using the primer sequences in Table 1. All mRNA levels were normalized against β‐actin mRNA levels, and the 2^−∆∆CT^ method was utilized to compute relative gene expression levels.

**TABLE 1:** Primer sequences.

Primer	Sequence
β‐Actin forward	5′‐CCCATCTATGAGGGTTACGC‐3′
β‐Actin reverse	5′‐TTTAATGTCACGCACGATTC‐3′
CASP‐3 forward	5′‐GAGCTGGACTGCGGTATTGAG‐3′
CASP‐3 reverse	5′‐AACCATGACCCGTCCCTTGA‐3′
LC3 forward	5′‐CATGCCGTCCGAGAAGACCT‐3′
LC3 reverse	5′‐GATGAGCCGGACATCTTCCACT‐3′
BECN1 forward	5′‐TGAGGAATGGAGGGGTCTAA‐3′
BECN1 reverse	5′‐TGGGCTGTGGTAAGTAATGG‐3′
BCL‐2 forward	5′‐CGACCTCTGTTTGATTTCTCCTG‐3′
BCL‐2 reverse	5′‐CTTTTCATATTTGTTTGGGGC‐3′

### 2.7 Enzyme‐Linked Immunosorbent Assay

To determine the levels of apoptosis (CASP‐3 and BCL‐2) and autophagy (LC3 and BECN1) markers, different commercial ELISA kits were purchased. The level of the mentioned markers was measured according to the instructions given by the manufacturer.

### 2.8 Statistical Analysis

The mean ± the standard error of the mean is used to report the data. The Kolmogorov–Smirnov test was employed to assess the normality of the data. The Kruskal–Wallis test was used for conducting the comparisons between groups, followed by a post hoc Mann–Whitney *U* test. Statistical analyses were performed using SPSS software (version 23.0 for Windows). A *p* < 0.05 was deemed statistically significant.

## 3 Results

### 3.1 Morin Ameliorated Ovariectomy‐Caused Weight Gain

**FIGURE 1:**
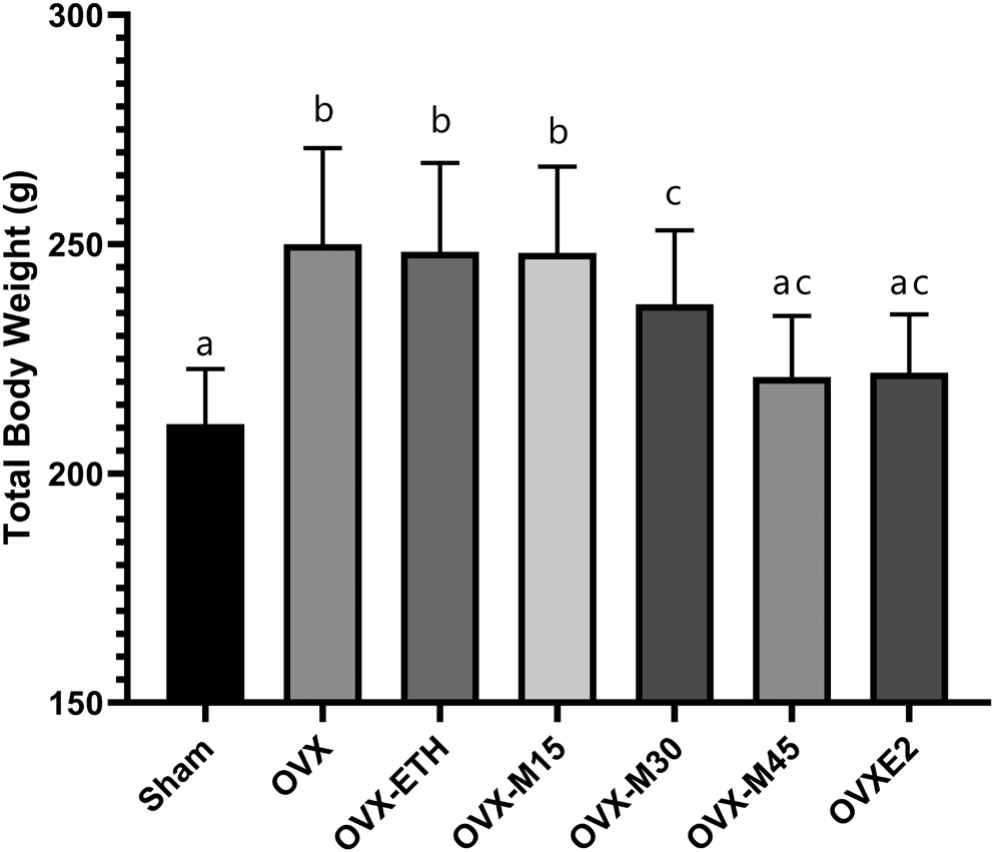
The measurement of total body weight. Ovariectomy caused a significant weight gain; however, morin at doses of 30 and 45 mg/kg/day and E2 ameliorated OVX‐induced weight gain. The results of the statistical analysis are indicated as lowercase letters on the bars, where different letters indicate a significant difference, whereas similar letters indicate no significant difference. *p* < 0.05 was considered significant.

The findings revealed that ovariectomy caused a notable rise in the total body weight of the subjects, as compared to the Sham group (Figure 1). Similarly, a significant increase was obtained when OVX‐ETH and OVX‐M15 animals were compared to the Sham group (*p* < 0.001). However, morin treatment with a dose of 45 mg/kg/day and 10 μg/kg/day of E2 restored ovariectomy‐caused weight gain (*p* < 0.0001).

### 3.2 Morin Restored the Bone Histoarchitecture of OVX Animals

**FIGURE 2:**
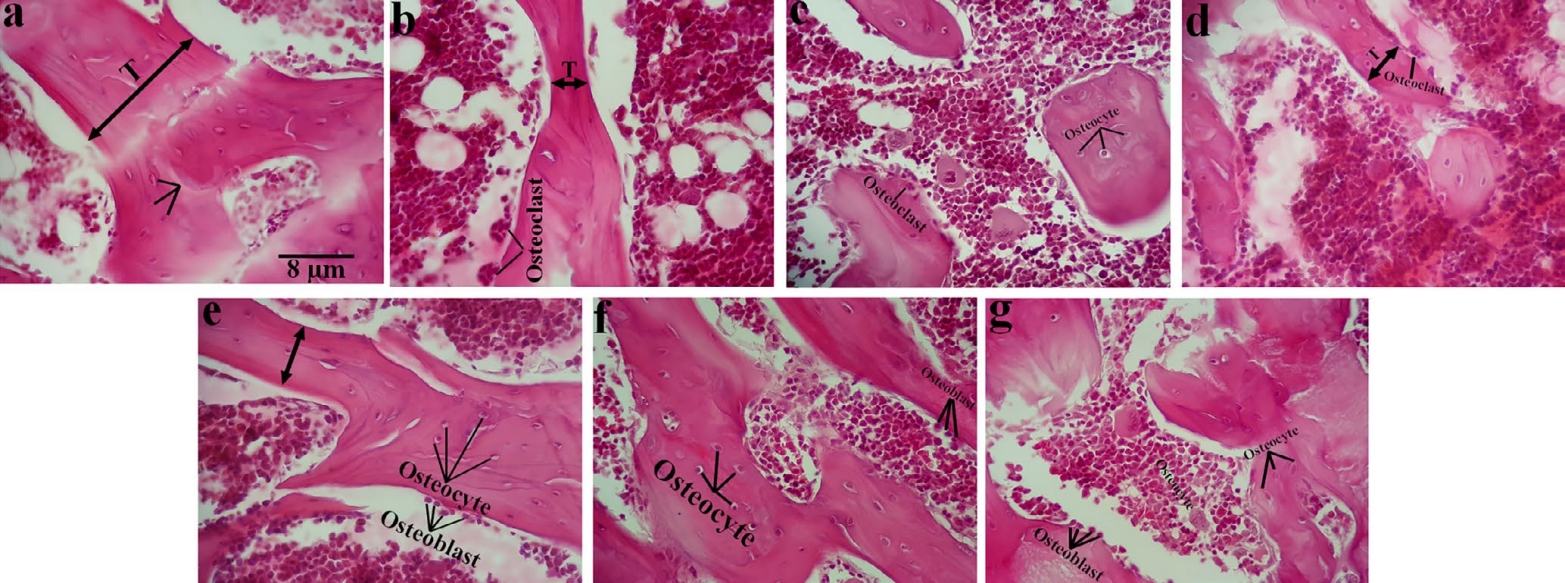
The histomorphology of studied groups. The figure compares the histoarchitecture of bone tissue between Sham (a), OVX (b), OVX‐ETH (c), OVX‐M15 (d), OVX‐M30 (e), OVX‐M45 (f), and OVXE2 (g) groups. H&E staining was performed. The scale bar is 30 μm.

**FIGURE 3:**
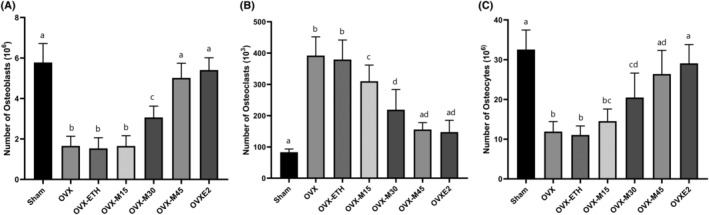
The comparison of the number of osteoblasts (A), osteoclasts (B), and osteocytes (C). OVX decreased the number of osteoblasts and osteocytes while increasing the number of osteoclasts. Morin restored the number of bone cells comparable to E2. Similar letters on the bars represent no significant difference between the studied groups. *p* < 0.05 was considered significant.

Microscopically, ovariectomy caused a significant bone injury, while morin and E2 were able to restore the tibia histoarchitecture in OVX animals (Figure 2). Stereologically, the study revealed that ovariectomy led to a notable reduction in the count of osteocytes and osteoblasts while significantly increasing the number of osteoclasts, especially when compared to the Sham controls. Nevertheless, OVX‐M45 and OVXE2 groups revealed a significant difference when compared to OVX animals regarding the number of bone cells (Figure 3).

### 3.3 Morin Alleviated Ovariectomy Effects on Tibial Weight and Volume

**FIGURE 4:**
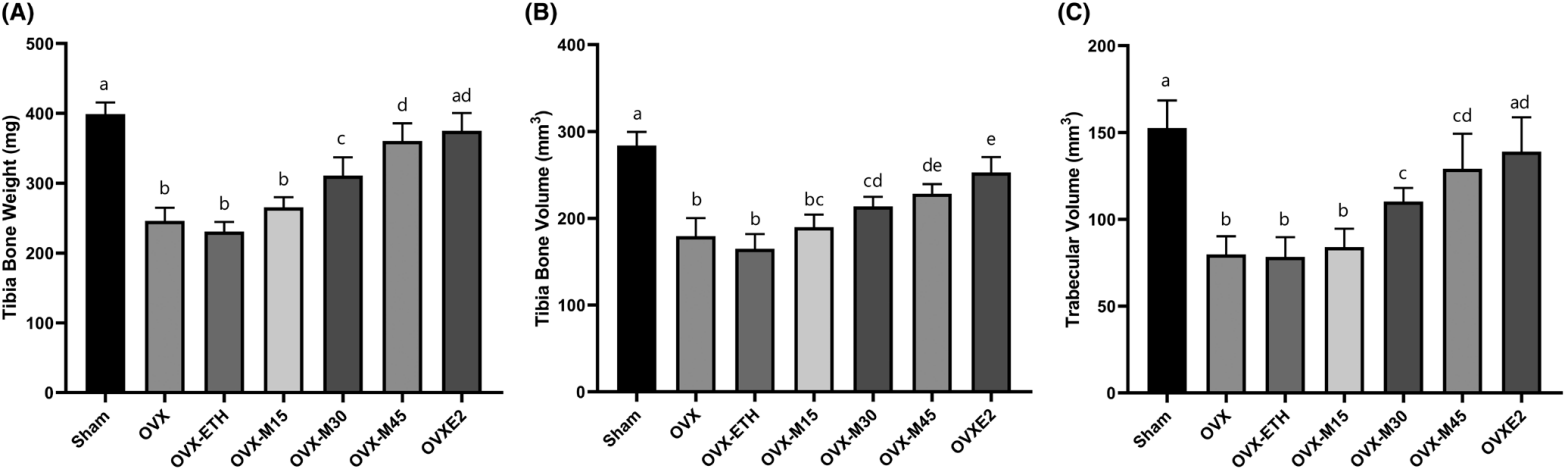
The weight (A) and volume (B) of the tibia and trabeculae volume (C). Although OVX decreased the weight and volume of bone‐related structures, morin represented ameliorative properties. Similar letters on the bars represent no significant difference between the studied groups. *p* < 0.05 was considered significant.

The study's results clearly showed that ovariectomy led to a notable reduction in both the tibia weight (Figure 4A) and tibia volume (Figure 4B) (*p* < 0.001). Moreover, the treatments OVX‐ETH and OVX‐M15 also exhibited a significant reduction in tibia volume and weight compared to the sham controls (*p* < 0.001). Interestingly, the administration of morin at doses of 30 and 45 mg/kg/day and 10 μg/kg/day of E2 resulted in a substantial increase in tibia weight and volume when compared to the OVX group (*p* < 0.05). Nevertheless, both of the mentioned doses of morin showed a significant difference with Sham controls regarding the tibia weight and volume, too (*p* < 0.05).

### 3.4 Morin Restored the Impact of Ovariectomy on the Overall Volume of Bone Trabeculae

Figure 4C demonstrates the volume of the trabeculae in the tibia in the studied groups. The findings showed a significant decrease in trabecular volume in OVX, OVX‐ETH, and OVX‐M15 groups compared to Sham (*p* < 0.001). Meanwhile, doses of 30 and 45 mg/kg/day of morin caused a significant increase of 38.31% and 61.88% compared to OVX animals (*p* < 0.001). However, morin administration in all studied doses caused a significant difference with the Sham control group in terms of trabecular volume (*p* < 0.05).

### 3.5 Morin Improved Bone‐Related Biochemical Markers in OVX Rats

**FIGURE 5:**
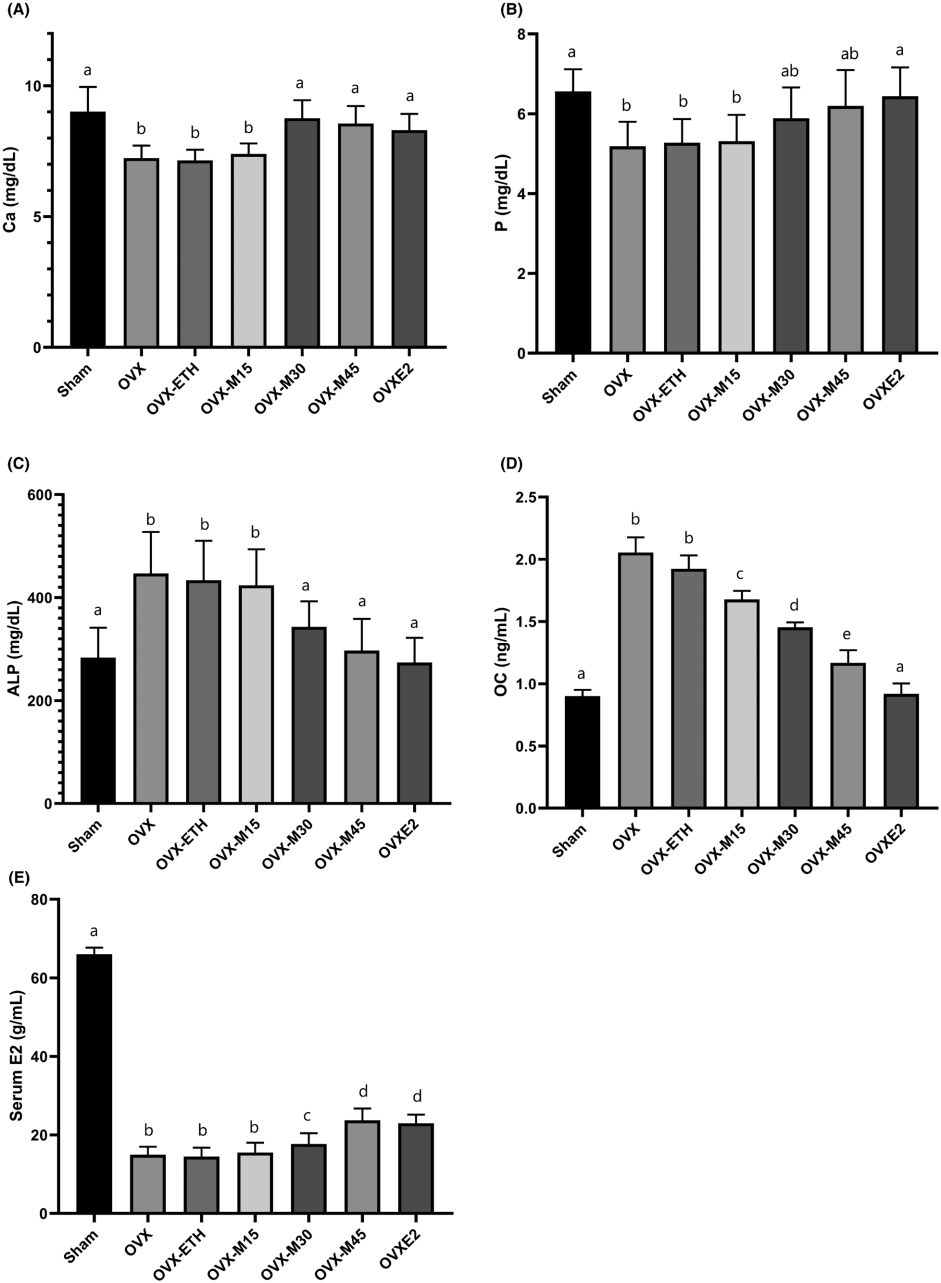
The analysis of bone‐related biochemical markers. OVX decreased Ca (A) and P (B) levels while the activity of ALP (C) and the level of OC (D) were increased. Moreover, OVX reduced serum levels of E2 (E). Similar letters on the bars represent no significant difference between the studied groups. *p* < 0.05 was considered significant.

The findings revealed that the serum levels of E2 were significantly reduced in OVX animals compared to the Sham group, while OVX‐M45 and OVXE2 caused a significant increase in E2 levels compared to OVX animals (*p* < 0.001, Figure 5). Moreover, the levels of Ca and P in OVX animals were significantly reduced compared to controls, whereas ALP and OC were remarkably elevated (*p* < 0.05). Nevertheless, OVX‐M30, OVX‐M45, and OVXE2 demonstrated a significant difference in comparison with OVX animals (*p* < 0.001).

### 3.6 Morin Suppressed Autophagy in Bone Tissue of OVX Rats

**FIGURE 6:**
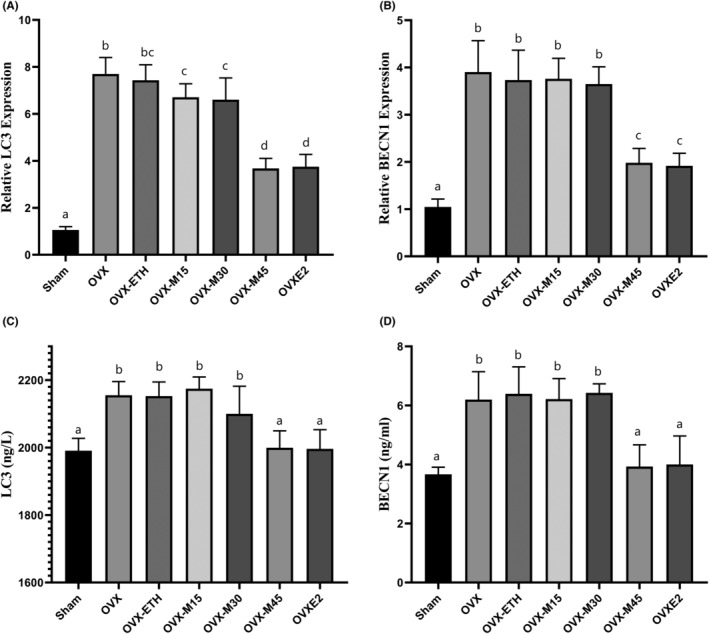
Morin suppressed OVX‐induced autophagy in bone tissue. The LC3 and BECN1 were measured in gene (A and B, respectively) and protein (C and D, respectively) levels. OVX promoted the levels of autophagy markers, while morin restored the elevated levels of LC3 and BECN1. Similar letters on the bars represent no significant difference between the studied groups. *p* < 0.05 was considered significant.

The present study benefited RT‐qPCR and ELISA techniques to evaluate markers related to autophagy at gene expression and protein levels (Figure 6). The findings showed that *LC3* and *BECN1* gene expression in the bone tissue of OVX rats increased by 7.29 times and 3.73 times, respectively, compared to the Sham group (*p* < 0.001). Moreover, the level of LC3 and BECN1 proteins in the OVX group showed a significant increase of 1.08 times and 1.69 times, respectively, when compared to the Sham (*p* < 0.001). Interestingly, a dosage of 45 mg/kg/day of Morin was effectively able to significantly restore the ovariectomy's effect on the expression of genes and the level of proteins related to autophagy. However, OVX‐M15 and OVX‐M30 animals represented no notable difference with the OVX group (*p* > 0.05).

### 3.7 Morin Inhibited Apoptosis in the Bone Tissue of OVX Animals

**FIGURE 7:**
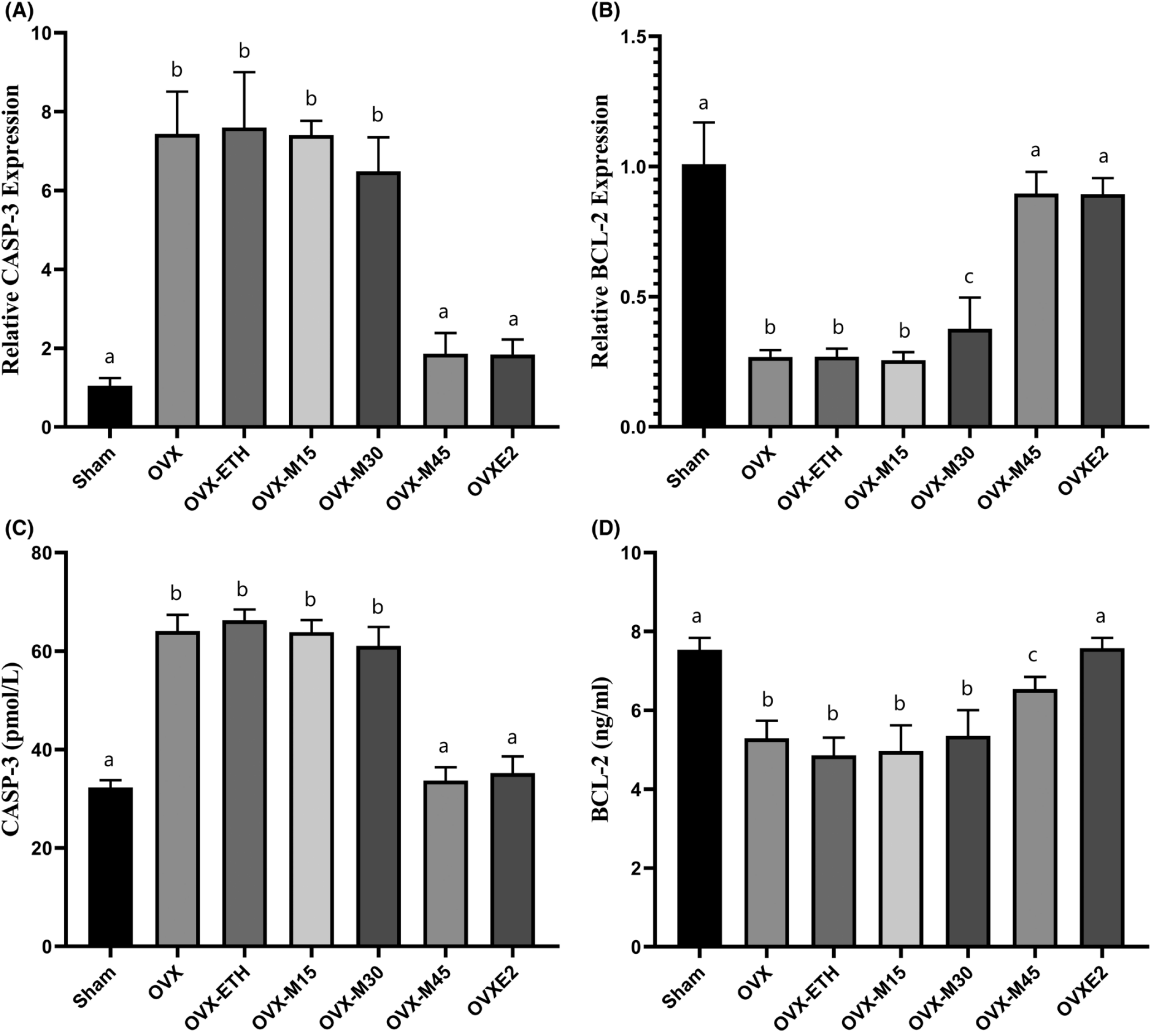
Morin inhibited apoptosis in the bone tissue of OVX animals. CASP‐3 and BCL‐2 were analyzed at gene (A and B, respectively) and protein (C and D, respectively) levels. Similar letters on the bars represent no significant difference between the studied groups. *p* < 0.05 was considered significant.

Expression of genes and protein levels involved in apoptosis were evaluated to investigate cell death in bone tissue (Figure 7). The findings revealed that the encoding gene expression and the protein level of CASP‐3 in the OVX group significantly increased by 7.11 times and 1.99 times (*p* < 0.001), respectively, compared to the Sham group. On the contrary, gene expression and protein level of BCL‐2 in the bone tissue of OVX group animals showed a significant decrease compared to the controls (*p* < 0.001). Meanwhile, the dose of 45 mg/kg/day of morin was able to significantly improve gene expression and protein levels of BCL‐2 (*p* < 0.05).

## 4 Discussion

OP remains one of the major concerns of the worldwide health system and the most common bone‐related metabolic disorder (Si et al. 2020), and efforts continue to find novel treatment approaches. The present study aimed to induce a model of OP in animals by ovariectomy and then evaluate the efficacy of morin compared to E2 on bone histomorphology, biochemical markers, and molecular mechanisms including autophagy and apoptosis.

Microscopically, the results of the current study demonstrated that the number of osteoblasts and osteocytes was significantly reduced in OVX animals, whereas ovariectomy led to a notable increase in the number of osteoclast cells, higher than those observed in the Sham group. Osteocytes are cells lying within the bone that is considered the main mechanoreceptors. Osteoblasts are specialized cells of primitive mesenchymal origin known as osteoprogenitor cells that mainly synthesize the components that constitute the extracellular matrix of bone and promote mineralization of the organic matrix (Mohamed 2008). Previous studies have assumed the reduction in the count of osteocytes and osteoblasts, alongside the escalation in osteoclasts, bone cells that degrade bone to initiate normal bone remodeling or cause bone loss in pathological states, as evidence of OP occurrence (Teitelbaum 2007; Wawrzyniak and Balawender 2022). The present findings revealed that morin in a dose of 45 mg/kg/day was able to significantly improve the effects of ovariectomy‐induced OP on bone cells and structures in an approach comparable to E2. Ongoing studies suggest phytochemicals as novel strategies for the treatment of OP, which may exert their potential properties by mimicking the E2 functions on its receptors, increasing the extra ovarian biosynthesis of the hormone, and changing the intracellular molecular mechanisms within bone tissue (Habauzit and Horcajada 2008; Sharma et al. 2023). Accordingly, the current findings demonstrated that morin significantly reduced the suppression of E2 synthesis after orectomy. Instead, some phytochemicals such as quercetin, which are described as a phytoestrogen, modulate the effects of this hormone on peripheral tissues such as bone by mimicking the action of E2 (Feng et al. 2024), while other compounds benefit different mechanisms to ameliorate the effects of E2 deficiency on bone structure (Ahmad Hairi, Jayusman, and Shuid 2023; Wei et al. 2023).

In addition to histomorphological and stereological analysis, the present study evaluated the levels of various bone‐related biochemical markers to assess morin effects on ovariectomy‐induced OP. The obtained results showed that ovariectomy caused a notable reduction in the serum levels of Ca and P, whereas OC level and ALP activity in the serum were significantly increased in OVX rats compared to Sham controls. Moreover, the dose of 45 mg/kg/day of morin indicated modulating effects on the level of mentioned biochemical markers in a manner comparable to E2. It is suggested by Albright's observations for the first time that E2 deficiency led to female OP (Prior 1990). It is documented that E2 prolongs the life span of osteoblasts and inhibits apoptosis of osteoblasts and osteocytes, where the E2 receptor is expressed abundantly (Bilezikian, Raisz, and Martin 2008). Previously, ALP are described as a marker of osteoblast activity, while OC is considered a main product of both osteocytes and osteoblasts. Similar to the results of the present study, Shapiro, Manola, and Leboff (2001) demonstrated that OP in women is accompanied by a sharp increase in OC and ALP levels, which may be an indicator of excessive activity of bone cells to compensate for the effects of OP. On the contrary, several studies have shown that OP caused by ovarian failure is followed by a reduction in one or both of the mentioned markers (Fan et al. 2018; Samare‐Najaf, Zal, and Safari 2020; Zhao et al. 2017). Nevertheless, the alteration in the level of bone markers along with the histomorphological findings can be attributed to the induction of OP caused by ovariectomy.

The abundant expression of E2 receptors on the surface of bone cells, especially osteocytes, causes anti‐apoptotic effects and, as a result, prolongs the lifespan of these cells, prevents bone resorption by osteoclasts, and protects against OP (Devi and Shanmugarajan 2023). Indeed, preventing the death of bone cells can be assumed to be the most important approach of E2 in the prevention of OP (Devi and Shanmugarajan 2023). Meanwhile, E2 deficiency in postmenopausal women or after ovarian failure caused by chemotherapy is associated with increased bone cell death and OP occurrence (Cheng, Chen, and Chen 2022). Accordingly, the present findings showed that both apoptosis and autophagy were significantly induced in the bone tissue of OVX rats. Autophagy has a dual approach in cells, as it may participate in maintaining intracellular homeostasis and survival by degrading unnecessary organelles/molecules (Samare‐Najaf, Neisy, et al. 2023a) or terminating cell fate by programmed death (Samare‐Najaf, Samareh, et al. 2023b). The findings of the current study showed that morin at a dose of 45 mg/kg/day inhibited cell death caused by apoptosis and autophagy in bone tissue comparable to E2. Studies on rodents have further revealed that the inhibition of autophagy, either through genetic manipulation or pharmacological interventions, can reduce osteoclast formation and bone resorption, which in turn may mitigate the effects of OP induced by ovariectomy (Lin et al. 2016). In fact, the ability of phytochemicals to maintain cell survival and homeostasis through the modulation of autophagy suggests these chemicals as nutrigenomics with high potential in the treatment of chronic diseases (Ragusa et al. 2023).

## 5 Conclusion

The present study established an animal model of OVX‐induced OP to evaluate the ameliorative properties of different doses of morin in comparison with E2. The findings showed that morin at a dose of 45 mg/kg/day was able to restore the effects of ovariectomy on bone histomorphology and improve the level of bone‐related biochemical biomarkers. The assessment of the level of apoptosis and autophagy in bone tissue showed that morin was able to suppress the programmed death of bone cells caused by ovariectomy. Taken together, the present findings suggest the promising potential of morin in confronting OP, although further studies are pivotally necessary.

## Author Contributions

Nan Jiang, Bo Qi, and Xinyu Fan contributed to the conception and design of the study and supervised the experiment; Ling Yao, Gang Li, and Xinyu Fan performed the experiments and interpreted data. All authors contributed to writing the manuscript and read and approved the final version of the manuscript.

## Ethics Statement

The animal research conducted in this study adhered strictly to the guidelines set forth by the National Institutes of Health for Laboratory Animal Care and Use. The study received approval from both the Institutional Animal Care and Use Committee and the local Ethics Committee of the University. To ensure the well‐being of the subjects, all animals were gradually acclimated to the laboratory environment 2 weeks prior to the initiation of treatments. Additionally, significant efforts were undertaken to alleviate animal discomfort and to minimize the number of experimental subjects utilized. The study was designed according to ARRIVE guidelines.

## Conflicts of Interest

The authors declare no conflicts of interest.

## Data Availability

The data are available upon reasonable request from the corresponding author.
